# The hidden burden of measles in Ethiopia: how distance to hospital shapes the disease mortality rate

**DOI:** 10.1186/s12916-018-1171-y

**Published:** 2018-10-18

**Authors:** Piero Poletti, Stefano Parlamento, Tafarraa Fayyisaa, Rattaa Feyyiss, Marta Lusiani, Ademe Tsegaye, Giulia Segafredo, Giovanni Putoto, Fabio Manenti, Stefano Merler

**Affiliations:** 1 Center for Information Technology, Fondazione Bruno Kessler, via Sommarive, 18, I-38123 Trento, Italy; 2 South West Shoa Zone Health Office, P.O. Box 253, Woliso, Oromia Ethiopia; 3 Doctors with Africa CUAMM, Woliso Hospital, P.O. Box 250, Woliso, Oromia Ethiopia; 4 Doctors with Africa CUAMM, via S. Francesco, 126, I-35121, Padova, Italy

**Keywords:** Mathematical model, Sub-Saharan Africa, Access to health care, Case fatality rate, Measles epidemic, Infectious diseases

## Abstract

**Background:**

A sequence of annual measles epidemics has been observed from January 2013 to April 2017 in the South West Shoa Zone of the Oromia Region, Ethiopia. We aimed at estimating the burden of disease in the affected area, taking into account inequalities in access to health care due to travel distances from the nearest hospital.

**Methods:**

We developed a dynamic transmission model calibrated on the time series of hospitalized measles cases. The model provided estimates of disease transmissibility and incidence at a population level. Model estimates were combined with a spatial analysis to quantify the hidden burden of disease and to identify spatial heterogeneities characterizing the effectiveness of the public health system in detecting severe measles infections and preventing deaths.

**Results:**

A total of 1819 case patients and 36 deaths were recorded at the hospital. The mean age was 6.0 years (range, 0–65). The estimated reproduction number was 16.5 (95% credible interval (CI) 14.5–18.3) with a cumulative disease incidence of 2.34% (95% CI 2.06–2.66). Three thousand eight hundred twenty-one (95% CI 1969–5671) severe cases, including 2337 (95% CI 716–4009) measles-related deaths, were estimated in the Woliso hospital’s catchment area (521,771 inhabitants). The case fatality rate was found to remarkably increase with travel distance from the nearest hospital: ranging from 0.6% to more than 19% at 20 km. Accordingly, hospital treatment prevented 1049 (95% CI 757–1342) deaths in the area.

**Conclusions:**

Spatial heterogeneity in the access to health care can dramatically affect the burden of measles disease in low-income settings. In sub-Saharan Africa, passive surveillance based on hospital admitted cases might miss up to 60% of severe cases and 98% of related deaths.

**Electronic supplementary material:**

The online version of this article (10.1186/s12916-018-1171-y) contains supplementary material, which is available to authorized users.

## Background

Measles is one of the most contagious vaccine-preventable viral diseases and represents an important cause of child mortality in sub-Saharan Africa [1, 2]. Despite considerable progress has been made during the last decade in measles mortality reduction [3], the persistent circulation of measles in the WHO African Region [1, 4–6] reflects the challenge of achieving sufficiently high herd immunity levels in areas with limited financial resources.

In low-income countries, a strong heterogeneity both in the measles case fatality rate [4^7^] and in the access to health care infrastructures has been widely documented [8–10], although rarely quantified and little understood [8–12].

In particular, some recent epidemiological studies, focusing on a variety of illness conditions, have shown that larger travel distances to large health care facilities are associated with lower hospital admission rates [8–10] and higher mortality [8, 9, 12]. However, these studies do not always differentiate between causes of hospitalization and death [11] and few recent works have documented measles mortality in sub-Saharan Africa [13]. As a matter of fact, the burden of disease is still often estimated on the basis of admitted hospital cases, representing a biased sample that does not reflect the severity of measles within the community [7].

In recent years, recurrent measles outbreaks, primarily affecting children below 5 years of age [1], have been reported in several areas of Ethiopia [1, 14], including the Oromia Region [4]. In Ethiopia, the national Expanded Programme on Immunization was established in 1980 and consists of the first dose of measles-containing vaccine (MCV1) administered at 9 months of age. Routine immunization of infants is supplemented by planned campaigns at 2- and 5-year intervals [3], aimed at increasing vaccination coverage and providing the opportunity for a second dose of vaccine to children who did not respond to the first one [3].

Here we analyze a sequence of annual measles epidemics, with 1819 hospitalized cases and 36 deaths, occurring from January 2013 to April 2017 in the South West Shoa Zone of the Oromia Region. Specifically, we describe the epidemiological characteristics of the observed epidemic, providing estimates of the disease transmissibility, incidence, and mortality at population level. In addition, we investigate the spatial heterogeneity characterizing both the detection and treatment of measles infections as a consequence of travel distance to the nearest hospital. The performed analysis highlights the potential hidden burden of disease caused by the heterogeneous access to primary health care in the region.

## Methods

### Study population and measles case patients

**Fig. 1:**
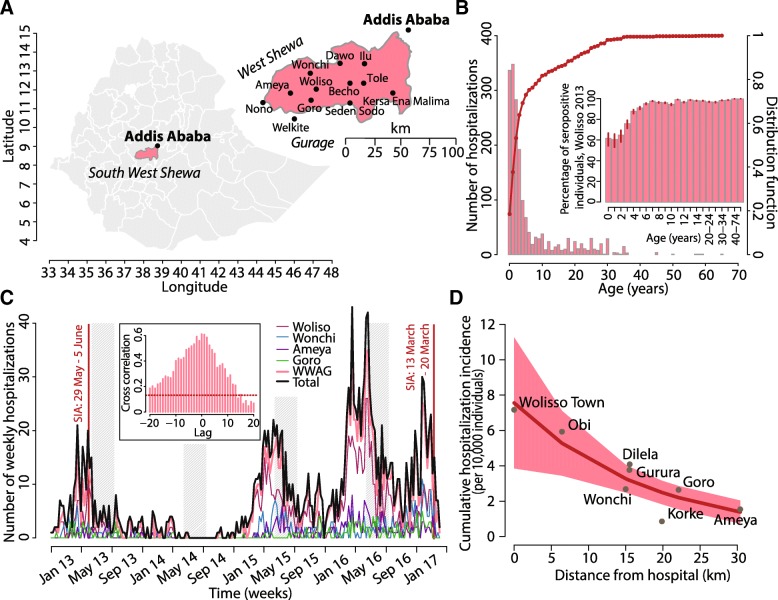
Epidemiological evidences: **a** Study area and spatial distribution of woredas. **b** Age distribution of measles patients hospitalized at the Woliso hospital between January 2013 and April 2017. The inset shows the estimated measles seroprevalence by age, as obtained on the basis of model estimates. **c** Time series of case patients recorded during the study period, overall, and in most affected woredas. The inset shows the cross correlation in the timing of epidemics in Woliso and most rural areas. **d** Cumulative incidence of hospitalizations per 10,000 individuals (h) by woreda/kebele and distance from Woliso hospital (d). The solid line represents estimates obtained by the negative binomial regression model; the shaded area represents 95% CI

This study was conducted in the South West Shoa Zone of the Oromia Region in Ethiopia (Fig. 1a), with an estimated population of 1,341,702 inhabitants in 2014, of whom 50.3% were men and 49.7% were women. The main hospital is located in Woliso town, 114 km southwest of the capital Addis Ababa, representing the nearest hospital for 521,771 individuals living within an area of 30 km radius from Woliso town (53,065 inhabitants). The hospital has 200 beds with an annual average bed-occupation rate of 84%; single-patient air-borne infection isolation rooms are not available in the hospital.

Data on age, sex, residence at woreda (i.e., district) and kebele (i.e., neighborhood) level, date of hospital admission, and death/discharge of measles case patients from 2013 to 2017 were obtained from the registers of Woliso hospital. Incidence of hospitalizations by woreda and kebele were calculated by assuming population projections for the 2014, based on the 2007 census conducted by the Central Statistical Agency of Ethiopia (Table 1) [15]. Travel distances to the Woliso hospital for different kebeles and woredas were obtained from administrative hospital records on distances of all health posts and largest villages distributed in the main hospital’s catchment area (see Table 1). The case fatality rate (CFR) for hospital admitted cases was calculated as the percentage of fatal cases among measles patients recorded. Routine vaccination coverage for this area was derived from administrative records: on average, 88% of children are routinely vaccinated against measles at 9 months of age. Two immunization campaigns were conducted in the area from May 29 to June 5, 2013, and from March 13 to March 20, 2017, targeting children 9–59 months of age [16]; the achieved vaccination coverage is unknown. In 2016, the vaccination status of case patients was assessed for 295 children in the age group 9 months to 5 years.

**Table 1:** Measles cases patients. Epidemiological characteristics of measles cases admitted to Woliso hospital (South West Shewa Zone, Oromia Region, Ethiopia) from January 1, 2013, to April 9, 2017

Mean age (years)	6.1 (SD 8.9; range 0–65)				
Deaths	36/1819 (2.0%)				
Females	855/1819 (47.0%)				
Vaccinated measles patients (2016)	120/295 (40.6%)				
Hospital main catchment area					
Woreda	Kebele	Patients	Deaths	Population	Distance (km)
Woliso	All kebeles	843	19	209,321	0–19.9
Woliso	Woliso town	379	8	52,849	0
Woliso	Obi	91	5	15.341	6.4
Woliso	Dilela	182	3	48,353	15.5
Woliso	Gurura	141	0	34,477	15.5
Woliso	Korke	50	3	58,301	19.9
Wonchi	All kebeles	296	1	110,275	15.0
Goro	All kebeles	147	3	55,640	22.1
Ameya	All kebeles	226	5	146,535	30.4
Woredas outside the hospital main catchment area					
Becho		41	3	91,116	33.7
Welkite (Gurage Zone)		31	1	55,097	40.1
Seden Sodo		19	1	82,969	45.0
Dawo		23	0	101,133	49.5
Ilu		19	0	75,326	55.9
Tole		48	0	75,438	73.6
Nono (West Shoa Zone)		45	1	108,356	82.7
Kersa Ena Malima		1	1	97,761	137.0
Other		80	1	Unknown	Unknown

Patients’ records related to different illness conditions recorded at the Woliso hospital between 2014 and 2016 were considered to estimate hospitalization incidence over time and to assess differences in the access to health care and related outcomes with respect to travel distances from the hospital.

Collected data consisted of routine health data and medical records, were encrypted and anonymous, and did not contain any information that might be used to identify individual patients; therefore, the study did not require informed consent.

### Synchrony of local epidemics

Synchrony in the timing of epidemics across different woredas was assessed by calculating the cross-correlation of time series at different time lags. The aim of this analysis is twofold: (i) to evaluate whether the observed seasonal pattern is an artifact of averaging asynchronous local epidemics and (ii) to support the hypothesis that observed measles cases were the result of a unique synchronous epidemic with similar epidemiological characteristics across different woredas.

### The modeling approach

The baseline analysis combines results of a dynamic transmission model, calibrated on the time series of hospitalized measles cases occurring between 2013 and 2017, with a spatial regression analysis, providing estimates of the measles hospitalization rate at different distances from the Woliso hospital. We restricted the analysis to measles cases from Woliso, Wonchi, Ameya, and Goro woredas, which represent the main hospital catchment area, consisting of 521,771 inhabitants and accounting for 83.1% of recorded case patients. Under the assumption of homogeneous mixing transmission, the baseline model provided estimates of the basic reproductive number (*R*_0_), the age-specific immunity profile, and the average measles incidence in the considered area. The estimated total number of infection cases in the population was disaggregated into smaller spatial units (woredas and kebeles), by assuming the same incidence rate across all spatial units and proportionally to the population size of each spatial unit. A regression model was applied to counts of observed hospitalized cases in each spatial unit to estimate the corresponding hospitalization rate; distance from the hospital was used as the independent variable and the estimated total number of cases in each spatial unit as offset. Obtained results were used to quantify the hidden burden of measles disease.

In the rest of this section, we detail the dynamic transmission model, the performed spatial analysis, how we calculated the hidden burden of disease, and the performed sensitivity analyses.

### The dynamic transmission model

Measles transmission dynamics between 2013 and 2017 is simulated through a deterministic, non-stationary, age-structured transmission model. In the model, the population is stratified in 86 1-year age classes, according to available data on the age distribution of the Ethiopian population in 2013 [17]. The crude birth rate of the population is 0.0325 years^−1^; individuals die according to age-specific mortality rates as reported between 2013 and 2015 and reflecting a crude mortality rate of 0.0083 days^−1^ [17]. The population of any age *a* is divided into five epidemiological classes: individuals protected by maternal antibodies (*M*_a_), susceptible individuals (*S*_a_), exposed individuals (*E*_a_), infectious individuals (*I*_a_), and individuals who acquired immunity against measles through either vaccination or natural infection (*R*_a_).

We assume that newborn individuals are protected against measles infection for 6 months on average by the passive transfer of maternal immunity [1], after which they become susceptible to the infection.

Susceptible individuals can acquire infection after contact with an infectious individual under the assumption of homogeneous mixing and become exposed without symptoms; at the end of the latent period, lasting 7.5 days on average, infectious individuals can transmit the infection for 6.5 days on average; the resulting generation time is 14 days [18]. After recovery, individuals are assumed to gain lifelong immunity. Newly infected individuals are hospitalized with a certain, age-independent, probability *p*_*h*_, representing the average hospitalization rate in the main hospital catchment area.

Seasonal variations in the transmission rate are considered: during school holidays, overlapping with the rainy season [14], the transmission rate is decreased by a factor *r*.

Routine vaccination of children is simulated at 9 months of age [3] with homogenous coverage across woredas at 88%. The latter estimate was obtained by administrative records on infant immunization occurring between 2013 and 2016 in the main hospital catchment area. Vaccine efficacy at the first dose of routine administration is assumed at 85% [19].

The follow-up campaigns conducted in 2013 (from May 29 to June 5) and in 2017 (from March 13 to March 20), targeting children 9–59 months of age [16], are also considered. The coverage of the 2013 supplementary immunization activities (SIAs), *c*_S_, was estimated among free model parameters. Vaccine efficacy during SIAs is assumed to be 95% [19].

Epidemiological transitions are described by the following system of ordinary differential equations:

$$ \left\{\begin{array}{ccc}{M_a}^{\prime}\left(\mathrm{t}\right)& =& bN(t)-\mu {M}_a(t)-\left({\varepsilon}_R{c}_R\left(t,a\right)+{\varepsilon}_S{c}_S\left(t,a\right)\right){M}_a(t)-d\left(t,a\right){M}_a(t)\\ {}{S_a}^{\prime }(t)& =& \mu {M}_a(t)-\left({\varepsilon}_R{c}_R\left(t,a\right)+{\varepsilon}_S{c}_S\left(t,a\right)\right){S}_a(t)-\beta (t){S}_a(t)I(t)/N(t)-d\left(t,a\right){S}_a(t)\\ {}{E_a}^{\prime }(t)& =& \beta (t){S}_a(t)I(t)/N(t)-\omega {E}_a(t)-d\left(t,a\right){E}_a(t)\\ {}{I_a}^{\prime }(t)& =& \omega {E}_a(t)-\gamma {I}_a(t)-d\left(t,a\right){I}_a(t)\\ {}{R_a}^{\prime }(t)& =& \gamma {I}_a(t)+\left({\varepsilon}_R{c}_R\left(t,a\right)+{\varepsilon}_S{c}_S\left(t,a\right)\right)\left({S}_a(t)+{M}_a(t)\right)-d\left(t,a\right){R}_a(t)\\ {}{H_a}^{\prime }(t)& =& {p}_h\omega {E}_a(t)\\ {}I(t)& =& {\sum}_{a=0}^{85}{I}_a(t)\\ {}H(t)& =& {\sum}_{a=0}^{85}{H}_a(t)\\ {}N(t)& =& {\sum}_{a=0}^{85}\left[{M}_a(t)+{S}_a(t)+{E}_a(t)+{I}_a(t)+{R}_a(t)\right]\end{array}\right. $$

where *t* represents time and *a* the individuals’ chronological age; *b(t)* and *d*(*t*,*a*) are the crude birth and the age-specific mortality rates at time *t*; 1/*μ* is the average duration of protection provided by maternal antibodies; 1/ *ω* and 1/*γ* are the average duration of the latent and the infectivity periods; *c*_*R*_(*t*, *a*) and *c*_*S*_(*t*, *a*) are the coverage associated with the first-dose routine vaccination and SIAs for individuals of age *a*, at time *t*; *ε*_*R*_ and *ε*_*S*_ represent the vaccine efficacy associated with routine vaccination of infants and SIAs. Specifically, *c*_*S*_ denotes the vaccinated fraction of individuals who were not yet immunized by natural infection or routine programs. *N*(*t*) and *H*(*t*) represent the total population of the hospital main catchment area and the cumulative number of hospitalized measles cases at time *t*; *p*_*h*_ is the fraction of measles infections that are hospitalized, and *β(t)* is the measles transmission rate defined as follows:

$$ \upbeta (t)=\left\{\begin{array}{c}\ r\ \upbeta,\ 1\mathrm{st}\ \mathrm{Jun}<\mathrm{t}<12\mathrm{th}\ \mathrm{Sep}\\ {}\upbeta, \kern0.5em \mathrm{otherwise}\end{array}\right. $$

At the end of the year, the chronological age of individuals is incremented by 1. The number of hospitalized measles cases in a time interval [*t*_1,_*t*_2_] is computed as *H*(*t*_2_) − *H*(*t*_1_).

Model estimates were obtained by simulating measles transmission between January 1, 2013, and March 20, 2017. Simulations are initialized on January 1, 2013. As the result of past natural infection and immunization campaigns, only a fraction *s*_0_ of the population is assumed to be susceptible to the infection. The age distribution of susceptibles at the beginning of 2013 was assumed to mirror the age distribution of hospitalized cases between January 2013 and March 2017. Specifically, the initial fraction of susceptible and immune individuals in each age group are *S*_*a*_(0) = *N*_*a*_*s*_0_*Z*_*a*_/${\sum}_{a=0}^{85}{Z}_a$ and *R*_*a*_(0) = *N*_*a*_ − *S*_*a*_(0), respectively, where *N*_*a*_ is the number of individuals of age *a* at the beginning of 2013 in Woliso, Ameya, Goro, and Wonchi [17] and *Z*_*a*_ is the observed total number of hospitalized measles cases of age *a*.

Free model parameters (*s*_0_, *β*, *r*_β_, *p*_h_, *c*_S_) were calibrated using a Markov Chain Monte Carlo (MCMC) approach based on the negative binomial likelihood of observing the weekly number of hospitalized case patients reported between January 1, 2013, and the beginning of the 2017 SIA. The scale parameter defining the negative binomial distribution was jointly estimated with other free parameters within the MCMC procedure. Details are provided in the Additional file 1.

### Reproduction number and disease elimination

The fundamental quantity regulating disease dynamics is the basic reproduction number (defined as *R*_0_ = 〈*β*〉/*γ*, where 〈*β*〉 is the average of *β*(*t*) over the year), which represents the average number of secondary infections in a fully susceptible population generated by a typical index case during the entire period of infectiousness. The larger the *R*_0_, the higher the disease transmissibility. If *R*_0_ > 1, the infection will be able to spread in a population. Otherwise, the infection will die out. For endemic diseases like measles, *R*_0_ provides insights into the proportion *p* of population to be successfully vaccinated to achieve disease elimination; the equation *p* = 1–1/*R*_0_ is widely accepted (e.g., [5, 18, 20]). For instance, if *R*_0_ = 10, at least 90% of children have to be routinely immunized to eliminate the disease.

### Spatial analysis

A negative binomial regression was used to study the relationship between incidence of hospitalization by kebeles/woredas and distance from Woliso hospital. Specifically, the observed number of hospitalized cases from each spatial unit is the response variable, the distance from the hospital is the independent variable, and the estimated total number of measles cases in each spatial unit (as estimated by the transmission model) is used as the offset.

Detailed origin of patients at the kebele level was used to better identify the travel distances for patients living within the Woliso woreda, where the hospital is located (Table 1).

In the negative binomial regression, we assume that counts of hospitalized cases *h*_*i*_ (the response variable) associated with a given location *i* are distributed as a negative binomial of mean *μ*_*i*_ determined by the number of infection in the location *c*_*i*_ (the offset) and the distance of location from the hospital *d*_*i*_ (the regressor) as follows:

$$ {\mu}_i=\exp \left(\ln \left({c}_i\right)+{b}_1+{b}_2{d}_i\right) $$

where *b*_1_, *b*_2_ are unknown parameters that are estimated from the observed hospitalized cases *h*_*i*_.

In order to take into account the uncertainty on incidence estimates obtained with the dynamic model, 10,000 draws from the posterior distribution of incidence estimates associated with 10,000 samples of the posterior distribution of free model parameters were considered to generate a distribution of regression model fits. Obtained results therefore account for the combined uncertainty due to the regression model and the dynamic transmission model.

We investigate the spatial variation in the incidence of hospitalized patients in the population as a consequence of different illness conditions. The aim is to characterize the relationship between hospitalization and distance from the hospital. The relative risk of being hospitalized at different distances from the hospital was computed by considering the incidence of hospitalization in each kebele/woreda divided by the incidence of hospitalized cases from Woliso town. The relative risk was fitted by an exponential function using distance as the independent variable (i.e., by fitting a linear model to the logarithm of the relative risk without intercept). Finally, a proportional test was used to assess possible statistical differences in the case fatality rate at hospital between cases coming from different sites.

### The hidden burden of disease

Persons living in Woliso town do not have distance barriers to access to the Woliso hospital. The probability of severe disease after measles infection was therefore computed as the fraction of measles patients from Woliso town that have been hospitalized for two nights or more among all measles infections estimated by the transmission model for this spatial unit. For severe cases, we indicate here those cases that from a clinical point of view are physiologically unstable and require supportive care (fluid resuscitation, oxygen, etc.) that can be provided only inside a well-resourced hospital. The resulting probability of developing severe measles illness *p*^*s*^ was used in combination with the estimated number of measles infections at different kebeles and woredas *c*_*i*_ to estimate the potential number of severe cases occurring at different distances from the hospital as *p*^*s*^*c*_*i*_. For each considered spatial unit *i*, missed severe cases were computed as the difference between the estimated number of severe cases and the number of patients recorded at the hospital, namely ${m}_i^s={p}^s{c}_i-{h}_i$. Missed severe cases were considered untreated and counted as additional deaths. The overall number of deaths caused by measles was estimated as the sum of missed deaths and measles deaths observed among hospital admitted patients. Averted deaths due to hospital treatment were estimated by considering all severe cases *p*^*s*^*c*_*i*_ as counterfactual deaths that would have occurred in the absence of adequate treatment.

### Sensitivity analyses

A variety of sensitivity analyses were conducted to evaluate to what extent some crucial assumptions made in the above described analysis may affect the obtained results.

We evaluated whether the assumption of decreased transmissibility during school holidays (or rainy season) is necessary to explain the observed pattern, by fitting a model with constant transmission rate against the time series of measles hospitalized cases.

Since the fraction of immunized individuals during the SIA in 2013 is unknown, we also considered two alternative models with *c*_S_ = 0 (SIA not conducted in 2013 in the considered area) and *c*_S_ = 0.92 (the highest coverage reported for past campaigns, namely 92% [3]).

We explored whether the assumption of homogeneous mixing, consisting in applying the same transmission rate to all age groups, can affect the model ability in reproducing the observed epidemiological patterns. To do this, we fitted the time series of cases with a transmission model encoding age-specific contacts as recently estimated for Ethiopia by Prem et al. [21]. In this case, increased mixing in schools corresponds to higher transmission rate among school-age children.

Models’ performances were assessed through the Deviance Information Criterion (DIC).

A sensitivity analysis was also conducted by fitting a transmission model to the time series of measles cases observed in Woliso, Wonchi, Ameya, and Goro separately. Specifically, a single epidemic was simulated in the four woredas simultaneously, by assuming the same initial conditions and by assuming that populations from different locations mix homogeneously. All epidemiological parameters were assumed to be equal across different woredas, but a different hospitalization rate was considered for each woreda.

An additional sensitivity analysis was performed to test whether estimates on the spatial variation of the hospitalization rates change when patients recorded from all woredas of the South West Shoa Zone are considered or when patients’ sex is considered.

Finally, estimates on the overall number of measles deaths and on the overall case fatality rate were estimated by relaxing the assumption that all missed/untreated severe measles cases die.

Details are provided in Additional file 1.

## Results

### Measles case patients

A total of 1819 case patients were recorded in Woliso hospital from January 1, 2013, to April 9, 2017 (Table 1). Of these, 855 (47.0%) were female and 964 (53.0%) were male; 1512 patients (83.1%) were resident in the main hospital’s catchment area, consisting of Woliso, Wonchi, Goro, and Ameya woredas. The mean age was 6.0 years (range, 0–65); 1259 case patients (69.2%) were aged ≤ 4 years and 1486 (81.7%) were aged ≤ 10 years (Fig. 1b). Records obtained during 2016 show that vaccinated admitted cases between 9 months and 5 years of age were 40.6%. In sub-Saharan Africa, different immunization rates may correspond to rural and urban areas [22, 23]. However, by looking at the vaccination status of hospitalized measles cases, though only recorded for a small fraction of cases, we found that the fraction of vaccinated individuals among measles cases was not significantly different across woredas (proportional test *p* value, 0.663) and consistent with administrative records of routine coverage in the area (see Additional file 1). This simple analysis partially supports the assumption of homogeneous coverage in the main catchment area.

The CFR based on hospital admitted cases was 1.98% (36/1819, 95% credible interval (CI) 1.43–2.72). The mean age of fatal cases was 3.3 years (range, 0–30). The time series of case patients is shown in Fig. 1c. Epidemic peaks were observed in June of 2013, 2015, and 2016, with marked incidence decrease after closure of schools for holidays and at the beginning of rainy seasons. A much lower number of case patients was recorded in 2014. In 2017, the epidemic peak was observed in late winter with marked incidence decrease after the conducted SIA (13–20 March).

### Measles transmissibility and seasonal patterns in measles circulation

Simpler transmission models with *r* = 1, *c*_S_ = 0, or *c*_S_ = 0.92 and the one based on heterogeneous mixing by age were all ruled out by the DIC analysis. Best model performances were obtained with the baseline transmission model. Remarkably, even if based on the assumption of homogeneous mixing, the baseline transmission model well reproduced the number of measles cases observed over time, among different age groups: 0–6 years, 7–14 years, and > 15 years (details in Additional file 1). Interestingly, we found that considering different transmission rate by age groups, as a consequence of heterogeneous mixing by age, does not improve the model ability in reproducing the observed time series of measles cases. The average reproduction number estimated with the baseline transmission model was *R*_0_ = 16.5 (95% CI 14.5–18.3).

A strong seasonal pattern of transmission was consistently observed across the different woredas. Significant synchrony in the timing of epidemics in Woliso and most rural areas was observed (inset of Fig. 1c and Additional file 1), so that the observed seasonal pattern was not an artifact of averaging asynchronous local epidemics. Model estimates suggest an average decrease in the force of infection of 27.8% (95% CI 21.6–33.2) between June and September, corresponding to school holidays and the rainy season.

The estimated average hospitalization rate in the main hospital’s catchment area was 12.4% (95% CI 10.9–14.1), similar to results found in [24]. Accordingly, 12,194 infections (95% CI 10,723–13,872), corresponding to a disease incidence of 234 per 10,000 individuals (95% CI 206–266), may have occurred in the area from January 1, 2013, to March 13, 2017.

The coverage of the 2013 SIA among residual susceptible individuals was estimated to be 18.7% (95% CI 11.9–24.3). The percentage of susceptible individuals at the beginning of 2013 was estimated to be 6.5% (95% CI 6.0–7.3). By assuming that the age distribution of observed measles cases mirrored the distribution of susceptible individuals across different age segments, we estimated the corresponding age-specific immunity profile of the population. This analysis showed that about 40% of children aged ≤ 2 years were not immunized against measles, while less than 10% of individuals aged > 5 years were susceptible to measles (inset of Fig. 1b).

### Spatial analysis

**Fig. 2:**
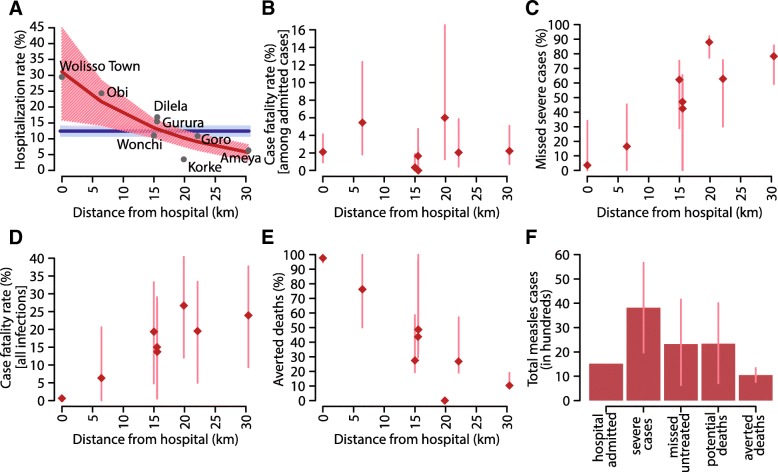
The hidden burden of measles disease. **a** Point estimates of the hospitalization rate at different distances from the Woliso hospital (in gray) and results from the negative binomial regression (mean in dark red and 95% CI in light red); estimates of the average hospitalization rate in the area as obtained with the transmission model are shown in blue (solid line represents the mean, shaded area represents 95% CI). **b** average CFR among hospital admitted cases across different sites (red diamonds); vertical bars represent 95% CI as obtained by exact binomial test. **c** Estimates of the proportion of untreated and missed severe cases over distance (diamonds represent the mean estimates; vertical bars represent 95% CI). **d** Estimates of the overall measles case fatality rate at different distances from the hospital; CFR is obtained as the fraction of estimated deaths over the estimated number of measles infections across different sites (diamonds represent the mean estimates; vertical bars represent 95% CI). **e** Estimated percentage of averted deaths due to hospital treatment as obtained by considering all severe cases as counterfactual deaths that would have occurred in the absence of adequate treatment (diamonds represent the mean estimates; vertical bars represent 95% CI). **f** Cumulative number of cases between 2013 and 2017 stratified in observed hospital admissions, estimated severe cases, missed untreated cases, overall potential deaths computed by assuming that all severe untreated cases died, and averted deaths due to hospital treatment (vertical bars represent 95% CI)

Differences in the case fatality rate among hospital admitted patients from different sites were not found statistically significant (see Fig. 2b). Significantly different cumulative incidences of hospitalizations by woreda and kebele were observed, with the largest values at 71 per 10,000 inhabitants in Woliso town (Fig. 1d). Cumulative incidence of hospitalizations by kebele/woreda was significantly correlated to travel distance from Woliso (Pearson *ρ* = − 0.90, *p* = 0.003) (Fig. 1d).

Estimated measles hospitalization rate dramatically decreases with travel distance from the hospital: from 31.0% (95% CI 15.9–45.0) in Woliso town to 5.7% (95% CI 3.0, 8.1) at 30 km from the hospital (Fig. 2a). Remarkably, similar estimates were obtained by fitting the transmission model to cases observed in Woliso (Woliso town and Obi, Dilela, Gurura, and Korke kebeles), Wonchi, Ameya, and Goro separately (see Additional file 1). In this case, estimates of woredas’ specific hospitalization rates range between 6.1% (95% CI 5.7–6.5) in Ameya and 15.9% (95% CI 15.0–17.0) in Woliso, with an average hospitalization rate in the hospital catchment area of 12.7% (95% CI 11.1–14.1) that is consistent with estimates obtained with the baseline model (see Additional file 1).

Similar results were also obtained when all woredas of the South West Shoa Zone were considered, although it is likely that measles cases occurring beyond 30 km from Woliso town have been partially detected, recovered, and treated in other health care facilities. A sensitivity analysis suggested that males had a higher access to health facilities with respect to females. However, the impact of distance on individuals’ access to care was found to not depend on the individual sex.

Interestingly, we found that the relative risk of hospitalization at the Woliso hospital associated with different illness conditions and health care treatments decreases with distance as well (see Additional file 1). These results suggest that the estimated decrease in measles hospitalization with the distance from the hospital is ascribable to inequalities in access to health care due to travel distances from the nearest hospital. These results, combined with those coming from the cross-correlation analysis of time series of cases from distinct woredas, suggest that observed measles cases were the result of a unique synchronous epidemic with similar epidemiological characteristics across different woredas. More details are provided in Additional file 1.

### The hidden burden of disease

The probability of severe illness once infected, based on measles inpatients from Woliso town, resulted in 0.30 (95% CI 0.16–0.43). The total number of severe measles cases in the Woliso hospital catchment area was consequently estimated to be 3821 (95% CI 1969–5671), only 1512 of which have been recorded among hospital admissions (Fig. 2c, f). By assuming that all untreated severe measles cases died, a total number of 2337 deaths (95% CI 716–4009) were estimated, 28 of which were detected at the hospital. Accordingly, 98% of deaths remained unobserved.

By estimating for each site the overall number of infected cases, the number of severe cases, and deaths, we found that the overall case fatality rate in the whole area (defined as the number of deaths per measles infection) might have been as high as 18.4% (95% CI 5.9–30.2).

Averted deaths due to hospitalization in the main hospital’s catchment area resulted to be 1049 (95% 757–1342). However, our results suggest that hospital effectiveness in preventing deaths dramatically reduces with travel distance from the hospital, becoming negligible beyond 20–30 km from the hospital (Fig. 2e). Our estimates suggest that the case fatality rate increases from 0.62% (95% CI 0.60–0.65) in Woliso town to more than 20%, on average, for sites that are more than 20 km far from the hospital (Fig. 2d).

**Fig. 3:**
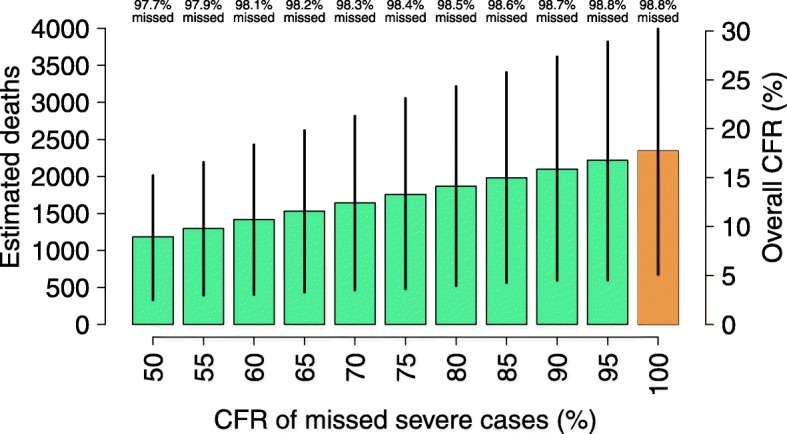
Sensitivity analysis. Total number of measles deaths (scaled on the left) and overall measles case fatality rate (scaled on the right) in the main hospital catchment area as estimated for different values of the fatality rate among severe cases that were not hospitalized. Estimates obtained with the baseline assumption are shown in orange. Vertical bars represent 95% of credible intervals. Percentages shown on top of the figure represent the estimated average proportions of deaths that were not reported at the hospital obtained with different values of the fatality rate among missed/untreated severe cases

The estimated number of deaths and the resulting CFR in the main catchment area decrease with the fatality rate assumed among severe cases that were not hospitalized (see Fig. 3). However, if only half of the severe cases that were not hospitalized are assumed to die, the estimated average number of measles deaths exceeds 1100, only 3% of which were recorded at the hospital; the estimated CFR among all infections results larger than 9% (see Fig. 3).

## Discussion

The epidemic in South West Shoa Zone highlights that measles still represents a major public health issue in Ethiopia. The synchrony of local epidemics and the consistent negative relationship between hospitalization incidence for different illness conditions and the distance from the referral hospital support the hypothesis of a large epidemic, spreading in the entire zone with similar transmission characteristics, but characterized by a significant heterogeneity in access to health care infrastructures.

The estimated average reproduction number of the observed epidemic was *R*_0_ = 16.5 (95% CI 14.5–18.3), slightly larger than values recently found for Niger (4.7–15.7) [20] and Zambia (12.6) [5]. Accordingly, the herd immune level required in the area to progress towards measles elimination is around 94%, far beyond possible achievements with routine administration of a single dose at 85% of vaccine efficacy [19, 25] and coverage at 88%. In particular, the estimated age-specific serological profile is consistent with estimates recently provided for Ethiopia [26], showing that, in 2015, 60% of susceptible individuals in Ethiopia were less than 5 years of age. These results suggest critically low immunization rates in recent birth cohorts.

Our analysis highlighted a significant reduction of measles transmission between June and September.

Such a reduction may reflect changes in contact rates induced by either school closure or rainfalls. Indeed, in the Oromia Region, school holidays occur during the rainy season [14]. Changes in measles transmission during this period was already observed in Ethiopia [14], and the decrease in measles circulation caused by rainfalls was suggested for other African countries [6], possibly due to relatively low connectivity or an increase in urban density during the dry season as a consequence of migration from agricultural areas. As already observed in Niger [6], the strong seasonality in measles transmission, combined with variations in vaccine uptake and in fertility rates may lead to erratic epidemiological patterns [27], characterized by frequent stochastic fadeouts, and irregular large epidemics. Occasional large outbreaks may be followed by years of very few cases, with inter-epidemic periods of unpredictable length and frequency, during which the high fertility characterizing the country can produce a fast, possibly unnoticed, recruitment of susceptible individuals [6, 26–28]. These considerations apply also to the South West Shoa Zone.

We found that the 2013 SIA might have reached less than 20% of residual susceptible individuals, which is much lower than the observed 75% reduction in the susceptible proportion produced by the first regional SIA conducted in southern Ethiopia in 1999 [29] and than the coverage levels estimated for SIAs conducted in other sub-Saharan countries (66–77%) [30]. The low impact of 2013 vaccination campaign with respect to past SIAs might have been influenced by problems in cold chain operations or vaccine maintenance [25] and the short duration of this campaign. However, the low impact of 2013 SIA may also reflect difficulties in immunizing individuals who escaped routine programs and past immunization efforts, especially through vaccination activities performed as a response strategy to ongoing epidemics [31].

Remarkably, we found that hospitalization rates and the effectiveness of passive surveillance based on hospital admissions, in both detecting measles and preventing measles-related deaths, dramatically decrease with travel distances from the hospital, becoming negligible beyond 20–30 km from the hospital. In particular, our estimates suggest that measles hospitalization rate decreases by about 80% within a 30-km travel distance from the hospital. These results are consistent with what observed in Kenya where all-cause admission rates were found to decrease by 11–20% with every 5-km increase in distance from the hospital [10]. A decrease of hospital admissions with increasing distance from the hospital was also found when estimating the global and regional burden of severe acute lower respiratory infections [32].

The overall estimated cumulative incidence was 2.34% (95% CI 2.06–2.66) of the population in less than 5 years. CFR among hospitalized cases was 1.98% (95% CI 1.43–2.72). However, while only 36 deaths were recorded at the hospital, the spatial epidemiological analysis performed highlighted that the observed epidemics may have caused about 2300 additional deaths, consisting of severe cases that did not received any hospital treatment. These results suggest that the overall case fatality rate among all measles infections might have been between 5 and 30%, significantly higher than published estimates for epidemics occurred in 2005–2006 in Niger, Chad, and Nigeria, namely 4.2–8.1% [13]. Obtained estimates for the measles CFR are consistent with those obtained for low-income countries during outbreaks occurring in isolated populations (above 15%) [7]. The assumed CFR among untreated measles cases essentially reflects our estimate of the percentage of most severe cases (around 30%), and it is in line with estimates of measles CFR in Ethiopia dating back to more than 30 years ago (around 27%) [7]. Estimates obtained on the total number of deaths and on the overall case fatality rate strongly depend on the assumption that all unobserved severe measles cases died. On the one hand, this represents a worst-case scenario. On the other hand, it is worth considering that cases here defined as severe are those with critical complications requiring to occupy, for two or more consecutive nights, one out of the 200 beds of a hospital in Ethiopia serving a potential catchment area of roughly 1.3 Million people and representing the closest well-resourced heath facility that can provide adequate treatments and supportive care for 521,771 inhabitants.

Obtained results are supported by spatial trends we identified in the relative risk of being hospitalized as a consequence of other illness conditions (see Additional file 1) and are consistent with what observed in previous studies on a variety of illness conditions [10, 22]. The role of distance as a barrier to health care access and affecting individuals’ mortality has been well documented by recent population-based studies [8, 9], although most of them do not differentiate between causes of death [11] and between levels of care available in facilities [11], and none of these are focused on measles. In particular, a cross-sectional survey recently conducted in Ethiopia highlighted that children who lived more than 30 km from the health center had a two- to threefold greater risk of death than children who lived near to the health center [8]. Similar results were found when considering either traveling distances or travel times [8]. In rural Tanzania, direct obstetric mortality was found to be four times higher at 35 km from hospital [11]. Finally, geographical clusters of acute abdominal conditions in India were found to have a nine times higher mortality rate and significantly greater distance to a well-resourced hospital [12].

All these epidemiological evidences suggest that what was observed for measles in the South West Shoa zone may likely affect other diseases and characterize other low-income settings of sub-Saharan Africa. Obtained results highlight that epidemiological estimates, based on hospitalization records only, may dramatically underestimate the burden of measles and should be carefully considered to design adequate and effective surveillance activities. More, in general, as already suggested in [10, 11], disease burden estimates based on hospital data may be strongly affected by distance from the hospital, although the amount of underestimation of disease burden may differ by disease [10, 11] and region considered.

The analysis has several limitations that should be considered in interpreting the results. The most important ones relate to the short observational period, the limited area considered, and the difficult task of quantifying unobserved severe measles cases. In particular, we assume that severe cases occurring within the main hospital’s catchment area that have not been reported at the Woliso hospital were not treated at all for measles disease. Although past studies have not found any association between child mortality and distance to small health facilities (e.g., health posts) [8], most severe infections might have seek treatment at hospitals that are more distant than the Woliso one. In addition, factors other than distance such as individual sex, age, family’s income, and geographical heterogeneity in incidence levels of comorbidities and social support provided to families might have strongly affected the access to health care and the disease outcome of patients coming from different locations [9]. Finally, misclassification of measles patients may always occur [7]. These limitations make it particularly difficult to reliably quantify untreated cases and estimate their fatality rate and the number of measles deaths, especially in absolute terms [7]. Other limitations of the proposed approach are determined by the lack of suitable data to model heterogeneous vaccination coverage within the hospital main catchment area, possible changes in the measles hospitalization rates over time, variations in the individual transmission rate of hospitalized cases, and seasonal variations of the population density as a consequence of migration flows between rural and urban areas.

## Conclusions

The carried out analysis represents a first attempt to investigate the impact of spatial heterogeneity in hospital accessibility on measles epidemiology, to quantify the hidden burden of measles in low-income settings, and to assess the effect of hospitalization in preventing death from severe measles disease. Epidemiological patterns identified through the performed analysis should be tested in other settings and may strongly depend on both levels of care available in health facilities [11] and infection rates in the considered community. If similar results will be confirmed, geographical heterogeneity in the hospitalization rates should be taken into account when estimating the burden of diseases and the effectiveness of the public healthcare system [7].

## Additional file

Additional file 1: Supporting material. Model details, sensitivity analysis, and additional results. (PDF 8654 kb)

## Abbreviations

CFR: Case fatality rate
CI: Credible interval
DIC: Deviance Information Criterion
MCMC: Markov Chain Monte Carlo
MCV1: First dose of measles-containing vaccine
*R*_0_: Basic reproductive number
SIA: Supplementary immunization activity
WHO: World Health Organization
