# Supplementary material for: The hidden burden of measles in Ethiopia: how distance to hospital shapes the disease mortality rate
Source: BMC Med. 2018 Oct 18;16:177. doi: 10.1186/s12916-018-1171-y (PMC6193289; doi:10.1186/s12916-018-1171-y)
Supplement: Supplementary file 1 — Supporting material. Model details, sensitivity analysis, and additional results. (PDF 8654 kb) [file 12916_2018_1171_MOESM1_ESM.pdf]

The hidden burden of measles in Ethiopia: how distance to hospital shapes the disease mortality rate

Piero Poletti[1,#], Stefano Parlamento[1], Tafarraa Fayyisaa [2], Rattaa Feyyiss[2], Marta Lusiani[3], Ademe Tsegaye[3], Giulia Segafredo[4], Giovanni Putoto[4], Fabio Manenti[4], Stefano Merler[1]
[1] Bruno Kessler Foundation, Trento, Italy
[2] South West Shoa Zone Health Office, Ethiopia
[3] Doctors with Africa Cuamm, Woliso Hospital, Ethiopia
[4] Doctors with Africa Cuamm, Padova, Italy

[#] Corresponding author: poletti@fbk.eu

Correspondence to: Piero Poletti, Center for Information Technology, Fondazione Bruno Kessler, via Sommarive, 18 I-20123 Trento, Italy poletti@fbk.eu

Supplementary Material

Contents

1. Spatial synchrony in the observed epidemics
2. Immunization rates across different woredas
3. Calibration of the transmission model
4. Alternative dynamic transmission models
5. Model selection and additional results
6. Alternative spatial regression models
7. The relationship between hospitalization and distrance from the hospital
8. References

1. Spatial synchrony in the observed epidemics

Spatial synchrony in the timing of epidemics observed across different woredas was assessed by computing the cross-correlation at different time lags of time series of cases associated with different areas of provenience. Temporal patterns in the number of measles admitted cases coming from the most affected woredas were compared: Woliso, Amaya, Goro, and Wonchi (Figure S1). Specifically, obtained results show that the pair-wise cross correlation between time series of cases occurring in two different woredas is always higher when no or negligible time lags are considered. This result indicates a significant synchrony in the timing of epidemics occurring across different geographical areas of the South West Shoa Zone.

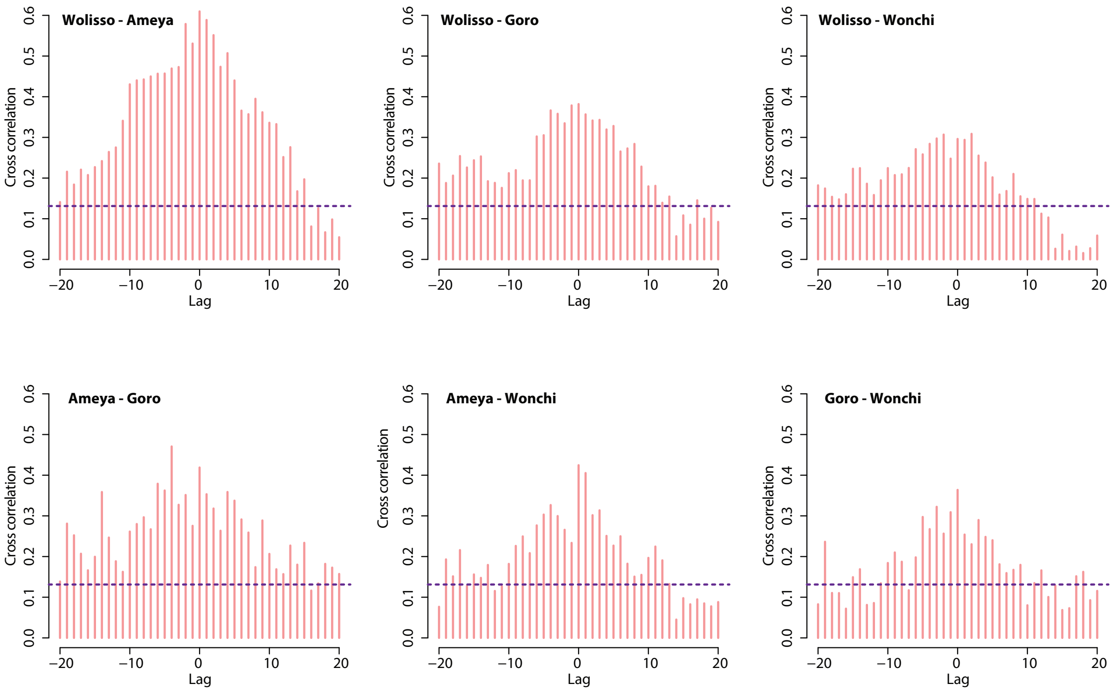

Figure S1. Cross correlation at different time lags (in weeks) of time series of hospitalized measles cases in most affected woredas.

2. Immunization rates across different woredas

In the analysis proposed in the main text, we assume homogeneous vaccination coverage across different locations within the hospital main catchment area.
In order to give some insights on potential differences in vaccine uptake levels across different woredas, we performed a simple analysis based on 295 measles cases hospitalized during 2016, for which the vaccination status was recorded (see Table 1). Interestingly, by neglecting individuals whose vaccination status is unknown, the fraction of vaccinated individuals among measles hospitalized cases were found to be not significantly different (proportional test p-value: 0.41) across woredas (Amaya, Goro, Wolisso, and Wonchi). Estimates of this fraction, denoted hereafter with P, are shown in Figure S2A. Confidence intervals on P were obtained using exact binomial test.

Woreda	Sample size	Vaccinated cases	Unvaccinated cases	Unknown vaccination status
Ameya	53	21	29	3
Goro	20	5	12	3
Woliso	171	76	86	9
Wonchi	20	9	9	2
Other	31	9	19	3
All	295	120	155	20

Table 1. Vaccination status recorded in a subset of measles hospitalized cases during 2016

The woredas-specific fractions of vaccinated cases among hospitalized measles cases were used to infer a rough estimate of the expected coverage levels across different woredas. In particular, we can assume that in each population of size N, I_n individuals has experienced measles natural infection, gaining life-long immunity against measles. Individuals who have never experienced measles infection can be therefore defined as $Y=N-I_n$.

In the absence of measles transmission, by assuming a vaccine efficacy e and constant vaccination coverage c on Y, Y can be stratified in three mutually exclusive classes:

- $I_v= Y\, c\, e$ denoting individuals who have been vaccinated and developed immunity against the infection as a consequence of vaccination.
- $S_v = Y\, c\, (1\text{-}e)$ representing individuals who have been vaccinated, but are still susceptible as a consequence of vaccine failure
- $S_{nv} = Y\, (1\text{-}c)$ defining individuals who are susceptible because they have neither experienced natural infection nor have been vaccinated.

Note that $S_{v+}S_{nv}$ represents the total number of susceptibles in the considered population.

By assuming that, during a new emerging epidemic, all susceptible individuals are exposed to the same force of infection λ, we can write the proportion P of infected cases that have been previously vaccinated as

$$P= \lambda\, S_v/(\lambda\, S_v+ \lambda\, S_{nv})= c\, (1\text{-}e) / (1\text{-}ec)$$

Through simple calculations we can derive the coverage c as

$$c=P/ (1\text{-}e\text{+}eP)$$

Figure S2B shows estimates of coverage levels across different woredas as obtained by using the equation derived for c, the woredas-specific estimates of P based on data shown in Table 1, and by assuming an average efficacy of 85%.

Remarkably, estimates of coverage levels resulted approximately homogeneous across different woredas and consistent with the average coverage reported in the main catchment area, namely 88% (Figure S2B).

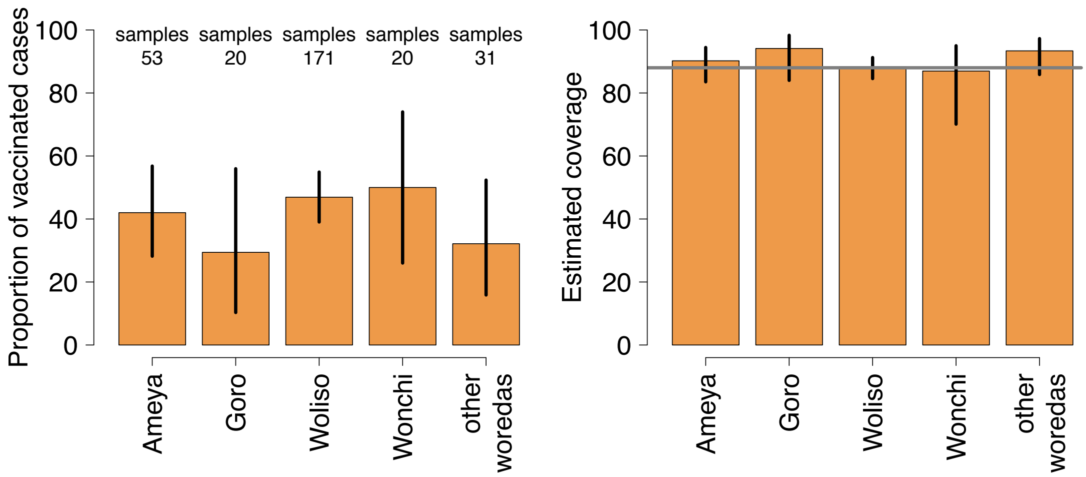

Figure S2. Proportions (*P*) of vaccinated patients among hospitalized measles cases as reported during 2016 across different woredas in the South West Shoa Zone (left panel, 95% of confidence intervals were computed using exact binomial test), and corresponding estimates of woredas specific coverage level (right panel), as obtained by using the formula *c=P/ (1-e+eP)* and a vaccine efficacy of 85%.

3. Calibration of the transmission model

Measles transmission dynamics between 2013 and 2017 is simulated using the model described in the main text. Initial conditions for the corresponding system of ordinary differential equations were set by assuming that:

1. the initial number of infectious individuals in the population is $I(0) = \frac{i_o}{p_h}$, where i_o is the number of hospital admitted cases in the first week of January 2013 and p_h is the unknown hospitalization rate;
2. initial infections are distributed across different ages proportionally to the age distribution of measles cases admitted to the hospital between January 1st 2013 and April 9th 2017, i.e. $I(0,a)=I(0)\, Z(a)/\sum_{a=0}^{85} Z(a)$, where $Z(a)$ is total number of hospitalized measles cases of age *a* as observed in the main hospital catchment area in the considered period of time;
3. the initial fraction of susceptible and immune individuals in each age group are $S(0,a)= N(a)\, s_0\, Z(a)/\sum_{a=0}^{85} Z(a)$ and $R(0,a)= N(a) - S(0,a)$ respectively, where N(a) is the number of individuals of age *a* at the beginning of the 2013 in Woliso, Amaya, Goro, and Wonchi [1] and s_0 denotes the unknown fraction of susceptible individuals at the beginning of the epidemic, i.e. on 1st Jan 2013.

The baseline transmission model has five free epidemiological parameter: β, r, p_h, c_S, s_0, where β represents the transmission rate during the dry season and when schools are open; r denotes the reduction in the force of infection (FOI) during school holidays/rainy seasons and c_S is the fraction of susceptible individuals who get vaccinated during SIAs.

Model calibration was carried out by performing a Bayesian statistical analysis of the reported number of hospitalized cases at the Woliso hospital between January 1st, 2013 and March 20th, 2016. Specifically, the posterior distribution of free model parameters was estimated by means of a Markov chain Monte Carlo (MCMC) approach, applied to the Negative Binomial likelihood of the weekly observed

hospitalized measles cases in the main hospital catchment area. In particular, we assume that hospitalized cases are distributed according to a Negative Binomial distribution, which is commonly expressed in terms of the mean m and dispersion parameter k. The probability mass function is:

$$P(x) \; = \; \frac{\Gamma(k+x)}{x!\,\Gamma(k)} \left(\frac{m}{m+k}\right)^{x} \left(1+\frac{m}{k}\right)^{-k}$$

where Γ denotes the standard Gamma function $\Gamma(\mathrm{n}+1)=\mathrm{n}!$.
The likelihood associated with a specific parameter set θ is therefore defined as:

$$\mathcal{L}(\vartheta, k|h) \; = \; \prod_{w} \frac{\Gamma(k+h(w))}{h(w)!\,\Gamma(k)} \left(\frac{m(w,\vartheta)}{m(w,\vartheta)+k}\right)^{h(w,)} \left(1+\frac{m(w,\vartheta)}{k}\right)^{-k}$$

where w runs over weeks between 1st January 2013 and 20th March 2017; $h(w)$ is the observed number of hospitalized individuals at week w; $m(w,\vartheta)$ is the estimated number of hospitalized measles cases during week w, as obtained by model simulation with a specific candidate parameter set ϑ. At each iteration, the algorithm evaluates the likelihood of a new candidate vector of parameters, which is accepted or not based on the standard Metropolis-Hastings algorithm. Uniform prior distributions are assumed for the free parameters and, at each iteration, each candidate value of a parameter is proposed from a normal distribution centered on the current value.
The scale parameter k defining the negative binomial distribution was jointly estimated with other free epidemiological parameters θ within the described algorithm.

Model estimates of the mean number of weekly-hospitalized measles cases over time are shown in Figure S3A. Remarkably, the baseline transmission model, although assuming homogeneous mixing by age, is able to well reproduce both the age distribution of observed cases (Figure S3B) and the number of weekly-hospitalized measles cases at different ages, namely between 0 and 6 years of age, between 7 and 14 years and older than 15 years of age (Figure S4A-C).

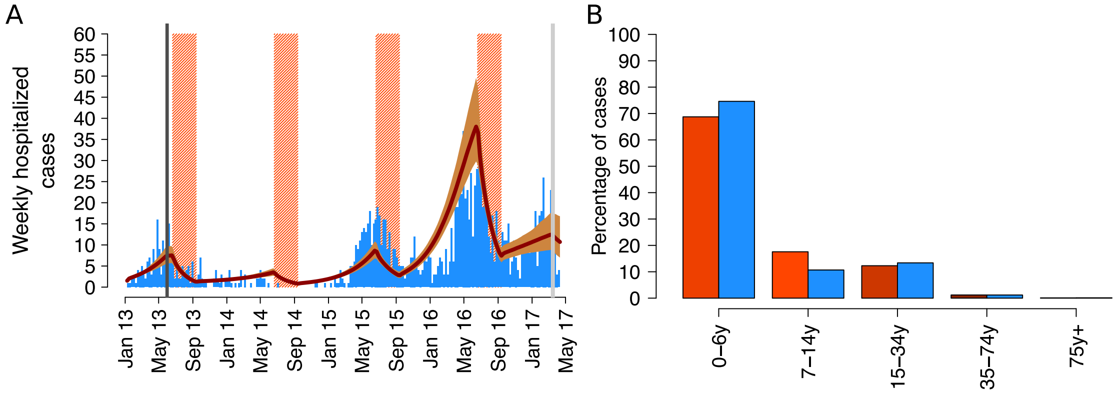

Figure S3 A) Estimated (mean in red and 95%CI in orange) and observed (blue bars) number of hospitalized measles cases between 2013 and 2017 in the main hospital catchment area, as obtained under the baseline scenario. Pink bars represents the period of time during which primary schools are closed, namely between 1st Jun and 12th Sep. B) Central model estimate of the age distribution of measles cases in the study area between 2013 and 2017 (in red) and age distribution of measles cases admitted to the hospital in the same period of time (in blue).

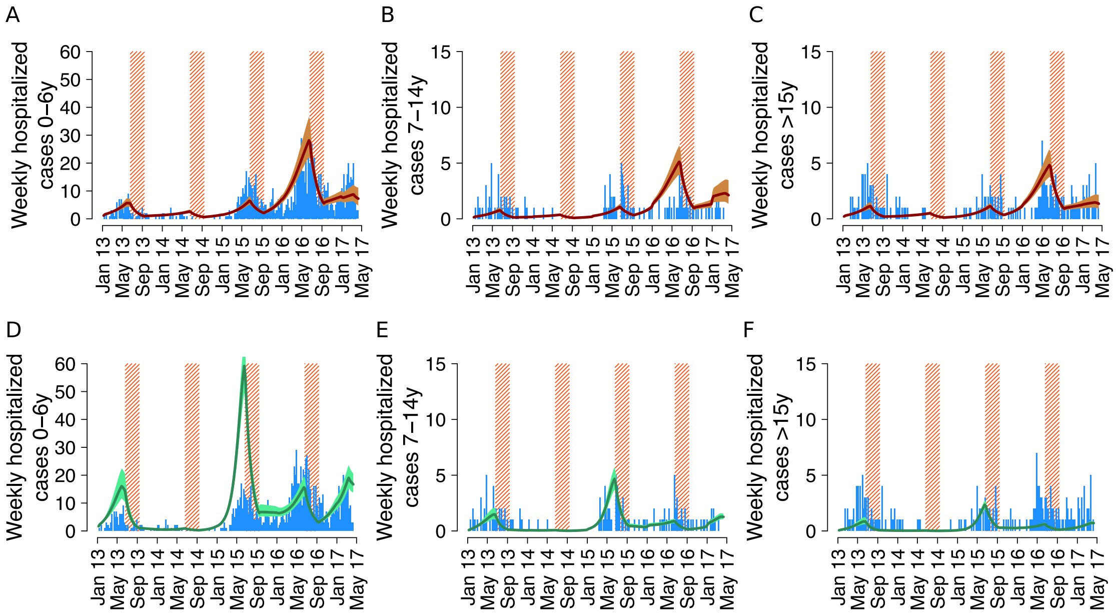

Figure S4 A) Estimated (mean in red and 95%CI in orange) and observed (blue bars) number of hospitalized measles cases between 2013 and 2017, among individuals younger than 6 years, as obtained under the baseline scenario. Pink bars represents the period of time during which primary schools are closed, namely between 1st Jun and 12th Sep. B) As A) but for individuals between 7 and 14 years of age. C) As A) but for individuals older than 15 years. D) As A) but as obtained with the model accounting for heterogeneous mixing by age. E) As D) but for individuals between 7 and 14 years of age. F) As D) but for individuals older than 15 years.

4. Alternative dynamic transmission models

A sensitivity analysis was conducted on different assumptions made in the baseline dynamic transmission model. The model was recalibrated by assuming either a constant transmission over the year or by fixing the coverage level for the SIA performed in 2013. For the latter case, we assumed that the SIA i) was not performed

in the considered area; ii) was performed with coverage at 92%, corresponding to the highest coverage reported for past campaigns [2]. In addition, we fitted the time series of cases with a transmission model encoding age-specific contact rates as recently estimated for Ethiopia by Prem et al. [3]. The latter analysis is detailed in the following sub-section and aimed at evaluating whether the assumption of homogeneous mixing affects the model ability in reproducing the observed time series of cases. Model selection was based on the analysis of the Deviance Information Criterion (DIC).

Finally we fitted the dynamic transmission model described in the main text to the time series of measles cases observed in Woliso, Wonchi, Ameya and Goro separately. Specifically, a single epidemic was simulated in the four woredas simultaneously, by assuming the same initial conditions and by assuming that populations from different locations mix homogeneously. All epidemiological parameters were assumed to be equal across different woredas, but a different hospitalization rate was considered for each woreda.
In this case, a different likelihood function was also considered therefore preventing a comparison between models based on DIC values. As a consequence, model performances were compared with results obtained with the baseline model using the root mean square error between the average model estimates and data on weekly hospitalized cases across different woredas. For the baseline model, the estimated numbers of hospitalized cases over time across different woredas were obtained using the average incidence estimated in the main hospital catchment area, multiplied by the woreda specific population size and the average hospitalization rate in each woreda, as obtained with the baseline regression analysis described in the main text.

Dynamic transmission model accounting for heterogeneous mixing by age

Measles transmission dynamics between 2013 and 2017 is simulated following the same approach used for the baseline transmission model. The unique difference characterizing the model based on heterogeneous mixing by age relies in the formulation of the age-specific force of infection $\lambda_a(t)$ acting on susceptible individuals of age a, namely S_a.
In the baseline model, we assume homogeneous mixing between individuals. This means that all susceptible individuals of age a are subject to an age independent force of infection defined as:

$$\lambda_a(t) = \beta(t)I(t)/N(t)$$

where I(t) and N(t) represent the total number of infectious individual and the population size at time t and $\beta(t)$ is the time-dependent transmission rate defined as

$$\beta(t) = \begin{cases} r\,\beta, & 1^{st}\,Jun\; < t < 12^{th}\,Sep \\ \beta, & otherwise \end{cases}$$

where β represents the transmission rate during the dry season and when schools are open, and r denotes the reduction in the force of infection (FOI) during school holidays/rainy seasons.
In the model based on heterogeneous mixing, an age-specific force of infection is defined as

$$\lambda_a(t) = \begin{cases} r\,\beta \sum_{a'=0}^{85} C_{a,a'} I_{a'}/N_{a'}, & 1^{st}\,Jun < t < 12^{th}\,Sep \\ \beta \sum_{a'=0}^{85} C_{a,a'} I_{a'}/N_{a'}, & otherwise \end{cases}$$

where $I_{a'}$ and $N_{a'}$ represent the total number of infectious individual and the population size in the age a' at time t; $C_{a,a'}$ is the average number of contacts per day that an individual of age a has with individuals of age a'. In the model $C_{a,a'}$ was assumed equal to the synthetic contact matrix recently estimated for Ethiopia [3].

In the model the population of any age a is divided into five epidemiological classes: individuals protected by maternal antibodies (M_a), susceptible individuals (S_a), exposed individuals (E_a), infectious individuals (I_a) and individuals who acquired immunity against measles either through vaccination or natural infection (R_a).

Simulated epidemiological transitions are described for each age class by the following system of ordinary differential equations:

$$\begin{cases} M_a'(t) &=& bN(t) - \mu M_a(t) - \big(\varepsilon_R c_R(t,a) + \varepsilon_S c_S(t,a)\big) M_a(t) - d(t,a) M_a(t) \\ S_a'(t) &=& \mu M_a(t) - \big(\varepsilon_R c_R(t,a) + \varepsilon_S c_S(t,a)\big) S_a(t) - \lambda_a(t) S_a(t) - d(t,a) S_a(t) \\ E_a'(t) &=& \lambda_a(t) S_a(t) - \omega E_a(t) - d(t,a) E_a(t) \\ I_a'(t) &=& \omega E_a(t) - \gamma I_a(t) - d(t,a) I_a(t) \\ R_a'(t) &=& \gamma I(t,a) + \big(\varepsilon_R c_R(t,a) + \varepsilon_S c_S(t,a)\big)\big(S_a(t) + M_a(t)\big) - d(t,a) R_a(t) \\ H_a'(t) &=& p_h \omega E(t,a) \\ I(t) &=& \sum_{a=0}^{85} I_a(t) \\ H(t) &=& \sum_{a=0}^{85} H_a(t) \\ N(t) &=& \sum_{a=0}^{85} [M_a(t) + S_a(t) + E_a(t) + I_a(t) + R_a(t)] \end{cases}$$

where *t* represents time and *a* the individuals' chronological age; *b(t)* are *d(t,a)* are the crude birth and the age specific mortality rates at time *t*; $1/\mu$ is the average duration of protection provided by maternal antibodies; $1/\omega$ and $1/\gamma$ are the average duration of the latent and the infectivity periods; $c_R(t,a)$ and $c_S(t,a)$ are the coverage associated with first dose routine vaccination and SIAs for individuals of age *a,* at time *t*; ε_R and ε_S represent the vaccine efficacy associated with routine vaccination of infants and SIAs. *N(t)* and *H(t)* represent the total population and the cumulative number of hospitalized measles cases at time *t*; p_h is the fraction of measles infections that are hospitalized. At the end of the year, the chronological age of individuals is incremented by 1.

The model is calibrated following the same procedure used for the baseline dynamic model. The basic reproductive number R_0 was instead computed using the Next Generation Matrix associated with a SEIR model where the transmission is driven by the contact matrix $C_{a,a'}$. Specifically, we computed $R_0 = \beta\rho(C_{a,a'})/\gamma$, where $\rho(C_{a,a'})$ is defined as the spectral radius of the contact matrix $C_{a,a'}$.

Dynamic transmission model with woredas specific hospitalization rates

In this model, the population of each woreda j and age a is divided into five epidemiological classes: individuals protected by maternal antibodies ($M_{a,j}$), susceptible individuals ($S_{a,j}$), exposed individuals ($E_{a,j}$), infectious individuals ($I_{a,j}$) and individuals who acquired immunity against measles either through vaccination or natural infection ($R_{a,j}$).
In each woreda measles transmission dynamics is simulated as in the baseline model described in the main text and by assuming that the population homogeneously mix across different woredas. The main difference characterizing the model fitted on woredas specific cases over time with respect to the baseline model relies on the definition of four woreda-specific hospitalization rates.

Simulated epidemiological transitions are described for each woreda and age class by the following system of ordinary differential equations:

$$\begin{cases} M'_{a,j}(t) &= bN_j(t) - \mu M_{a,j}(t) - (\varepsilon_R c_R(t,a) + \varepsilon_S c_S(t,a))M_{a,j}(t) - d(t,a)M_{a,j}(t) \\ S'_{a,j}(t) &= \mu M_{a,j}(t) - (\varepsilon_R c_R(t,a) + \varepsilon_S c_S(t,a))S_{a,j}(t) - \beta S_{a,j}(t)I(t)/N(t) - d(t,a)S_{a,j}(t) \\ E'_{a,j}(t) &= \beta S_{a,j}(t)I(t)/N(t) - \omega E_{a,j}(t) - d(t,a)E_{a,j}(t) \\ I'_{a,j}(t) &= \omega E_{a,j}(t) - \gamma I_{a,j}(t) - d(t,a)I_{a,j}(t) \\ R'_{a,j}(t) &= \gamma I_{a,j}(t) + (\varepsilon_R c_R(t,a) + \varepsilon_S c_S(t,a))\left(S_{a,j}(t) + M_{a,j}(t)\right) - d(t,a)R_{a,j}(t) \\ H'_{a,j}(t) &= p_{h,j}\omega E_{a,j}(t) \\ I(t) &= \sum_j \sum_{a=0}^{85} I_{a,j}(t) \\ H_j(t) &= \sum_{a=0}^{85} H_{a,j}(t) \\ N_j(t) &= \sum_{a=0}^{85} \left[M_{a,j}(t) + S_{a,j}(t) + E_{a,j}(t) + I_{a,j}(t) + R_{a,j}(t)\right] \\ N(t) &= \sum_j N_j(t) \end{cases}$$

where j is an index defining one of the four possible woredas, namely Woliso, Wonchi, Goro, Ameya; $N_j(t)$, $N(t)$ and $H_j(t)$ represent the total population in the woreda j, the total population in the main hospital catchment area and the cumulative number of hospitalized measles cases at time t in the woreda j, respectively; $p_{h,j}$ is the fraction of measles infections occurring in woreda j that are hospitalized at the Woliso hospital.

Model calibration was carried out by adopting the same approach used for other dynamic transmission models. However, in this case, free model parameters consist in four site independent free epidemiological parameters β, r, c_S, s_0, the scale parameter k defining the negative binomial distribution and four woredas specific hospitalization rate $p_{h,j}$. The posterior distribution of free parameters was estimated by means of a Markov chain Monte Carlo (MCMC) approach, applied to the product of the Negative Binomial likelihood associated with the weekly observed hospitalized measles cases in each of the four considered woredas.
The likelihood associated with a specific parameter set θ was therefore defined as:

$$\mathcal{L}(\vartheta, k|h_j) = \prod_j \prod_w \frac{\Gamma(k + h(w,j))}{h(w,j)!\,\Gamma(k)} \left(\frac{m(w,j,\vartheta)}{m(w,j,\vartheta) + k}\right)^{h(w,j)} \left(1 + \frac{m(w,j,\vartheta)}{k}\right)^{-k}$$

where j runs over the four woredas, w runs over weeks between 1st January 2013 and 20th March 2017; $h(w,j)$ is the observed number of hospitalized individuals at week w in site j; $m(w,\vartheta,j)$ is the estimated number of hospitalized measles cases during week w for the site j, as obtained by model simulation with a specific candidate parameter set ϑ.

5. Model selection and additional results

Table 2 shows DIC values associated with different modelling assumptions and the corresponding parameters' and epidemiological estimates, as obtained through the MCMC approach described above. Estimates obtained with different model assumptions are also displayed in Figure S5. For the sake of comparison, we computed the average hospitalization rate in the main hospital catchment area associated with the model fitted separately for each woreda. The latter was computed as the weighted sum of estimates obtained for the hospitalization rate in each woreda, using the woredas' population size as weights (see Figure S5).

	DIC	SIA coverage (%)	R_0	FOI reduction during summer (%)	Susceptible individuals in 2013 (%)	Hospitalization Rate (%)
Baseline model	1089	18.7 (11.9-24.3)	16.5 (14.5-18.3)	27.8 (21.6-33.2)	6.5 (6.0-7.3)	12.4 (10.9-14.1)
No seasonal forcing	1162	39.8 (32.3-45.1)	20.7 (18.6-22.0)	0	5.8 (5.4-6.3)	11.6 (11.0-14.4)
No SIA in 2013	1128	0	12.2 (11.0-13.6)	33.3 (26.6-39.2)	8.2 (7.4-9.2)	13.1 (10.9-15.0)
SIA coverage assumed	1257	92	69.6 (66.1-73.0)	23.1 (17.9-29.2)	1.8 (1.7-2.0)	31.8 (26.9-36.7)
Heterogeneous mixing by age	1127	19.4 (10.9-25.5)	11.0 (9.7-11.8)	32.1 (28.2-36.1)	2.2 (2.1-2.5)	12.2 (10.7-13.9)
Separate fit for each woreda	-	22.4 (17.5-28.0)	15.5 (14.0-17.1)	27.1 (22.3-31.6)	7.1 (6.5-7.8)	6.7 (5.5-7.8) in Ameya<br>12.4 (9.7-15.0) in Goro<br>16.3 (13.7-18.5) in Woliso<br>12.8 (10.7-14.7) in Wonchi

Table 2. Summary of model results as obtained under different modelling assumptions.

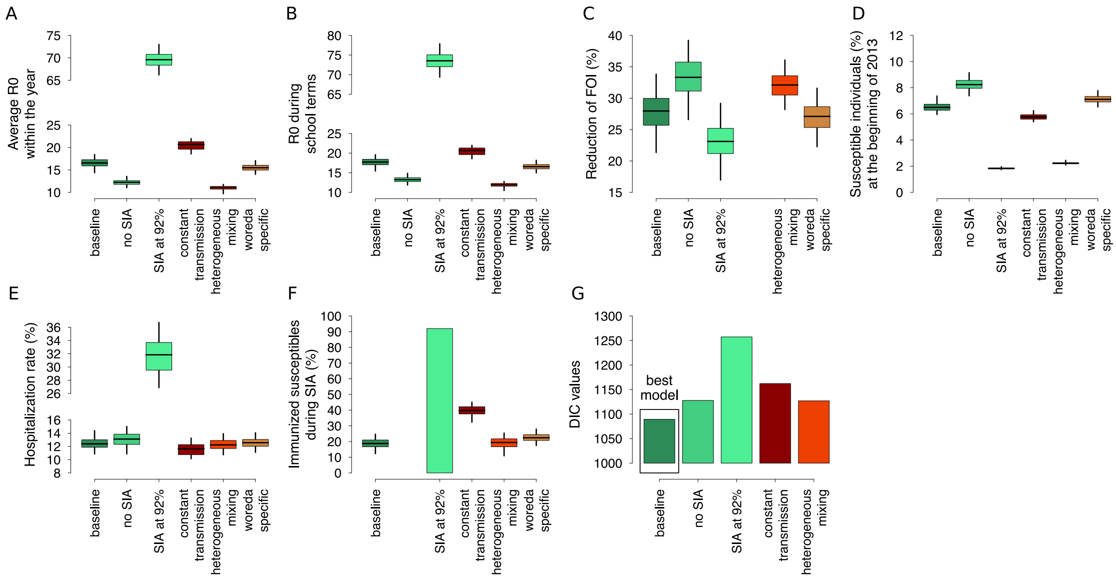

Figure S5 A) Estimates of R_0 obtained with the baseline scenario and with the alternative assumptions made for the sensitivity analysis. B) As A) but the estimated R_0 during the school terms (corresponding to the dry season). C) As A) but for the estimated reduction in force of infection during school holidays (r). D) As B) but for the estimated percentage of susceptible individuals in the population at the beginning of the epidemic (s_0). E) As B) but the estimated hospitalization rate (p_h). A weighted average is shown for the model based on woreda-specific hospitalization rates. F) As B) but for the estimated coverage level characterizing the 2013 SIA (c_s). G) DIC values associated with different modelling assumptions considered.

On the basis of DIC values, both the inclusion of a seasonal forcing in measles transmission and a parameter aimed at estimating the coverage associated with the 2013 SIA improve the model capability of reproducing the observed epidemiological patterns (see Table 1 and Figure S6). Specifically, in the absence of a seasonal variation in the transmission rate, the model fails to reproduce the decrease in the number of hospitalised measles cases observed during school holidays/rainy season (see Figure S6A). When the coverage of the 2013 SIA is assumed to be 92%, the model completely fails in reproducing the overall epidemic pattern observed between 2013 and 2017 (see Fig. S6B). However, by assuming that the 2013 SIA was not performed in the study area, model fit underestimates the observed increase of hospitalized cases occurring between January and May 2013 (see Figure S6C).
The obtained results therefore suggest that the 2013 SIA was at least partially effective in decreasing measles circulation between September 2013 and June 2015 and that a significant reduction in measles transmission regularly occur between June and September, possibly due to either school closure or rainfalls.
On the other hand, the inclusion of realistic age-specific contact patterns (stratified in 5-years age-bands) was ruled out by the analysis of DIC values associated with different models considered. Although age-specific contact patterns may be relevant when investigating the transmission of infectious diseases, 70% of reported infections were <4 years of age and the model based on heterogeneous mixing by age fails in reproducing the temporal series of hospitalized cases observed in the main hospital catchment area (Figure S6D). However, it is worth noting that the model based on heterogeneous mixing by age was informed with a synthetic contact matrix recently estimated for this country [3]. Indeed, data on age-specific contact patterns in Ethiopia are not yet available and the unique work providing some information on this is represented by a very recent study by Prem et al. [3], which provides only synthetic

contact matrices across different countries based of a statistical analysis of their demographic structure.
Finally, Figure S7 shows model estimates of the mean number of weekly-hospitalized measles cases over time across different woredas, as obtained by fitting cases from Woliso, Ameya, Goro and Wonchi, separately. The estimated posterior distributions of woredas specific hospitalization rates obtained with this model were compared with the woredas specific hospitalization rates obtained with the baseline regression analysis presented in the main text. More specifically, the hospitalization rate characterizing the Woliso woredas for the baseline regression analysis was computed as the weighted average of hospitalization rates estimated across all kebeles in the Woliso woreda, by assuming weights equal to kebeles specific population sizes. In addition, the average measles incidence estimated with the baseline transmission model was scaled to the woreda level, using woreda specific population sizes and hospitalization rate as resulting from the baseline regression model. This allowed us to compare the two models (separate fit vs baseline) in terms of the root mean square error (RMSE) between model estimates and observed data at the woreda level. Remarkably, negligible differences were found by using the two alternative modeling approaches (see Figure S7).

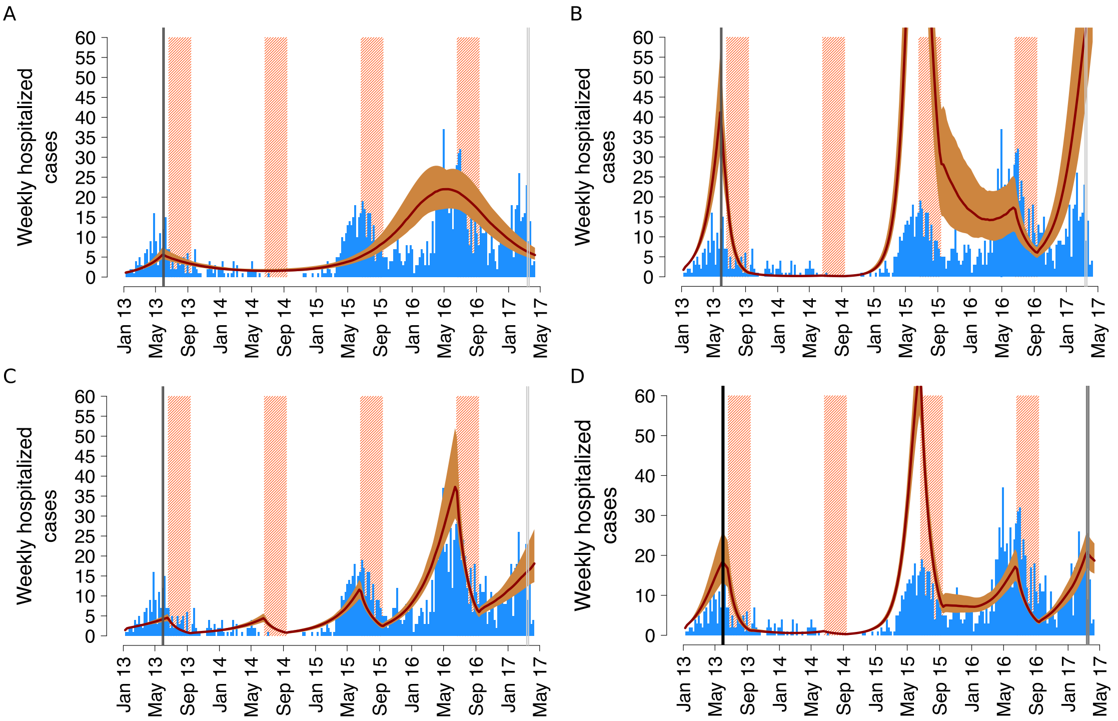

Figure S6 A) Estimated (mean in red and 95%CI in orange) and observed (blue bars) number of hospitalized measles cases between 2013 and 2017, as obtained by assuming a constant transmission rate over time. B) As A) but by assuming 92% coverage for the 2013 SIA. C) As A) but by assuming that the 2013 SIA was not performed in the study area. D) As A) but as obtained with the model accounting for heterogeneous mixing by age.

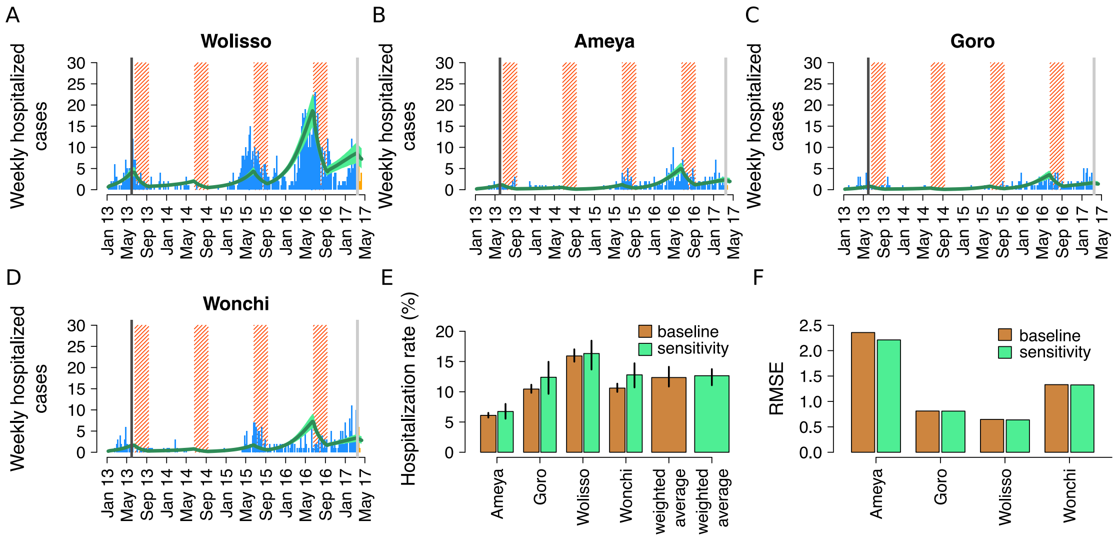

Figure S7. A) Estimated (mean in dark green and 95%CI in light green) and observed (blue bars) number of hospitalized measles cases between 2013 and 2017, among individuals of Woliso woreda, as obtained by fitting a model taking into account woreda-specific hospitalization rates. Pink bars represents school closures. B) As A) but for Ameya. C) As A) but for Goro. D) As A) but for Wonchi. E) Comparison between estimates of woreda-specific hospitalization rates as obtained by directly fitting the dynamic model against cases reported in Woliso, Ameya, Goro and Wonchi separately and as obtained with the baseline approach. F) Comparison of the root mean square error (RMSE) between model estimates and observed data obtained by directly fitting the dynamic model against cases reported in Woliso, Ameya, Goro and Wonchi separately or through the baseline approach.

6. Alternative spatial regression models

Alternative regression models were also considered to assess the robustness of results obtained in the main text. In particular, we performed a sensitivity analysis to investigate the spatial dependence in the hospitalization rates when patients recorded from all woredas of the South West Shoa Zone zone are considered. Although it is likely that measles cases occurring beyond 30km from Woliso town have been partially detected, recovered and treated in other health care facilities, our results suggest that the marked decrease of measles hospitalization rate with travel distance from the hospital do not critically depend on the selection of sites adopted in our baseline regression analysis (Figure S8).

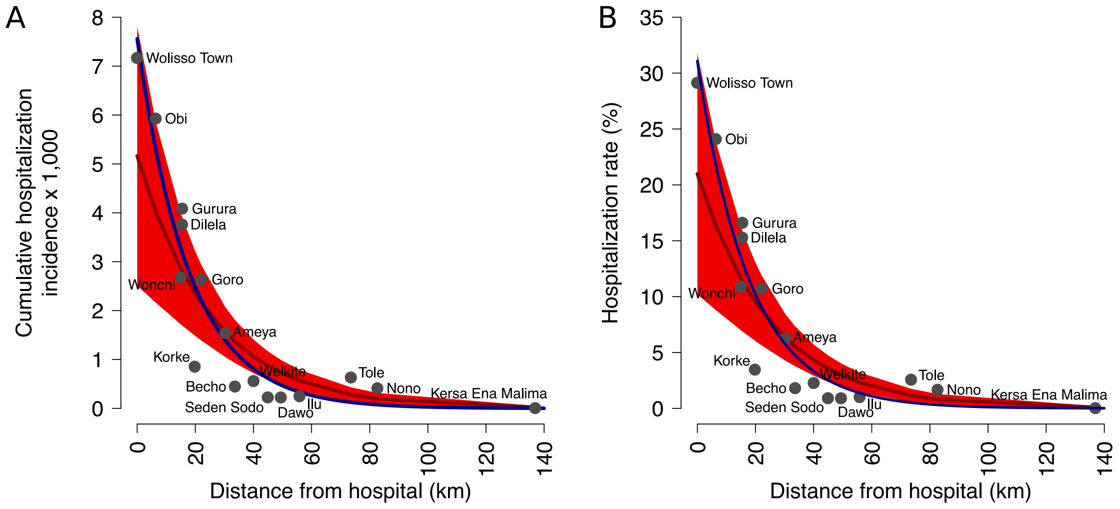

Figure S8 A) Cumulative incidence of hospitalizations per 1,000 individuals by distance from Woliso hospital in the entire South West Shoa zone. The red solid line represents estimate obtained by the negative binomial regression model applied to all sites in the South West Shoa zone; shaded area represents 95%CI. The blue solid line represents the mean estimate obtained by the negative binomial regression model applied only to the main hospital catchment area (baseline scenario in the main text). B) as A) but for the measles hospitalization rate.

We also assessed whether the identified spatial dependence of the measles hospitalization rate was dependent on sex of measles patients. This analysis was conducted by considering a separate regression analysis for male and female patients. As we do not have any information on the different coverage level by sex in the area, this analysis assumes that measles incidence was the same among males and females. Obtained results suggest a slightly higher measles hospitalization incidence among males. Although this result highlights a possibly different access to health facilities of males with respect to females, the observed changes in both the cumulative hospitalization incidence and the measles hospitalization rate strongly suggest that the role of travel distance in shaping individuals' access to care does not depend on the individual sex (Figure S9).

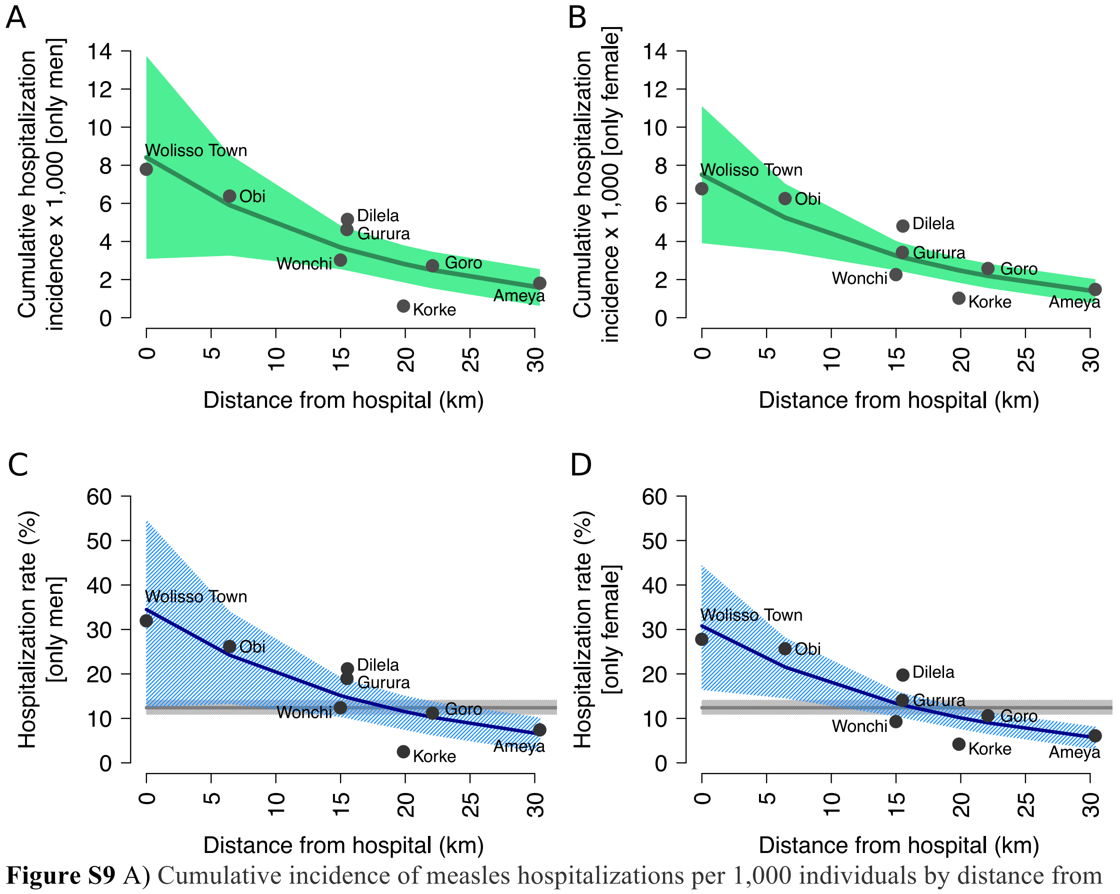

Figure S9 A) Cumulative incidence of measles hospitalizations per 1,000 individuals by distance from Woliso hospital as obtained when considering only male patients. The green solid line represents the mean estimate obtained by the negative binomial regression model applied to all sites in the main hospital catchment area; shaded area represents 95%CI. B) As A) but for females. C) As A) but for the measles hospitalization rate. D) As B) but for the measles hospitalization rate.

7. The relationship between hospitalization and distance from the hospital

Results obtained from the spatial analysis of measles hospital admissions at different distances are supported by spatial trends we identified in the relative risk of being hospitalized as a consequence of other illness conditions.
In particular, we investigated the risk ratio (RR) of being hospitalized for individuals living at a certain distance from the hospital with respect to those living in Woliso town.
Hospitalization causes considered in this analysis include - among others - other infectious diseases (e.g. malaria, TB, HIV), respiratory tract infections (RTI), traumas, and natural deliveries (see Figure S10). It is worth noting that hospitalization due to natural delivery consists of hospital admissions of patients that had not been affected by any complication raised neither post-partum nor during pregnancy.
In general, the risk ratio (RR) is defined by ratio of the probability of an event occurring in an exposed group to the probability of the event occurring in a comparison, non-exposed group. In our case, we computed the relative risk associated with a given distance d from the hospital as $RR(d) = p_h(d)/p_h(d=0)$, where: i) $p_h(d)$ is probability of being hospitalized at a certain distance and it is computed as the cumulative hospitalization incidence in the population living at distance d; ii) $p_h(d=0)$ denotes the probability of being hospitalized for individuals that are close to the hospital and it is computed as the cumulative hospitalization incidence in Woliso town. Cumulative hospitalization incidence rates were obtained dividing the number of cumulative hospitalized cases in a given site by the population size of the same site.
More specifically, we performed a linear regression analysis applied to the logarithm of the RR computed for different causes of hospitalization, by considering as the response variable the travel distance from the hospital (d) and assuming no intercept (i.e., $log\left(RR(d)\right) = \phi d$, where ϕ can be either positive or negative). The analysis can be interpreted as fitting the RR with an exponential function of distance, constrained to be 1 at 0km (i.e., $\mathrm{RR}(\mathrm{d}) = e^{\phi d}$).
Obtained results (Figure S10) highlight a marked decrease of the relative risk of hospitalization with distance from Woliso for all the causes of hospitalization considered but for malnutrition. Actually, for malnutrition an increase of RR with distance from the hospital is estimated. To some extent, this is something expected as malnutrition is much more prevalent in rural areas.
Interestingly, we found that the decrease in the relative risk of seeking hospitalization for measles as a function of distance is milder than what observed for other health outcomes (Figures S10 and S11). However, confidence intervals associated with different outcomes often intersect each other so that, for most of outcomes, no significant differences were found (Figure S11).

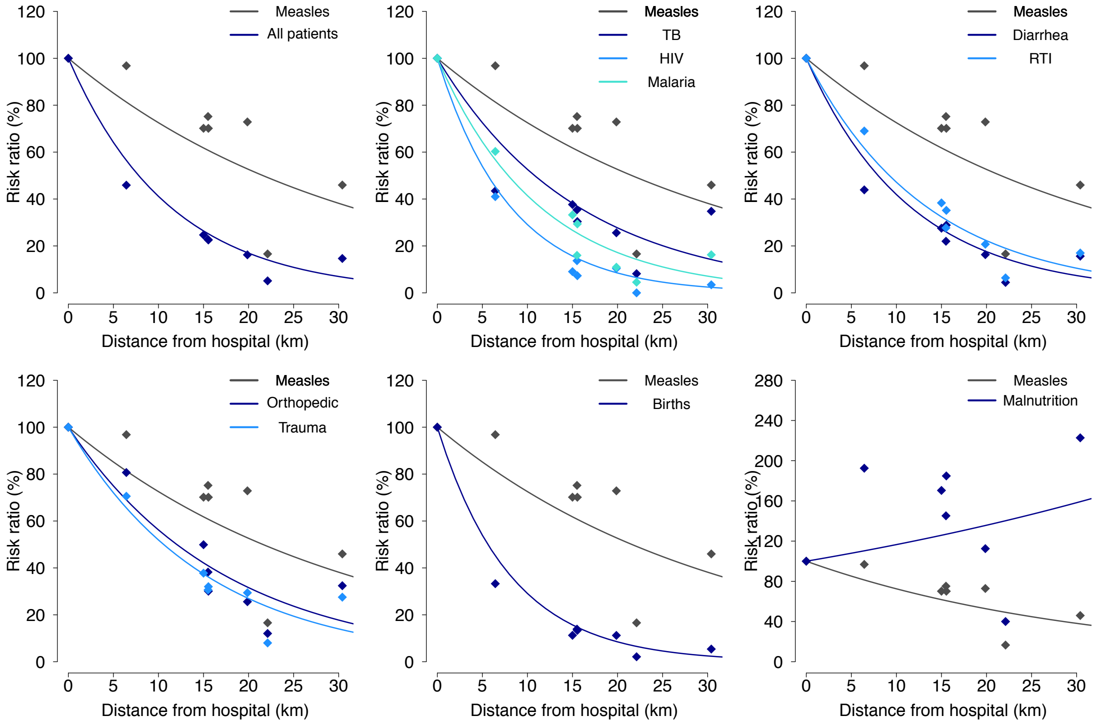

Figure S10 Points represent estimates of the relative risk (RR) of being hospitalized for different causes of hospitalization across different sites in the main hospital catchment area. Different colours are used for different hospitalization causes. Solid lines represent mean estimates obtained using a linear regression model applied to the corresponding log(RR) using distance as independent variable and assuming no intercept.

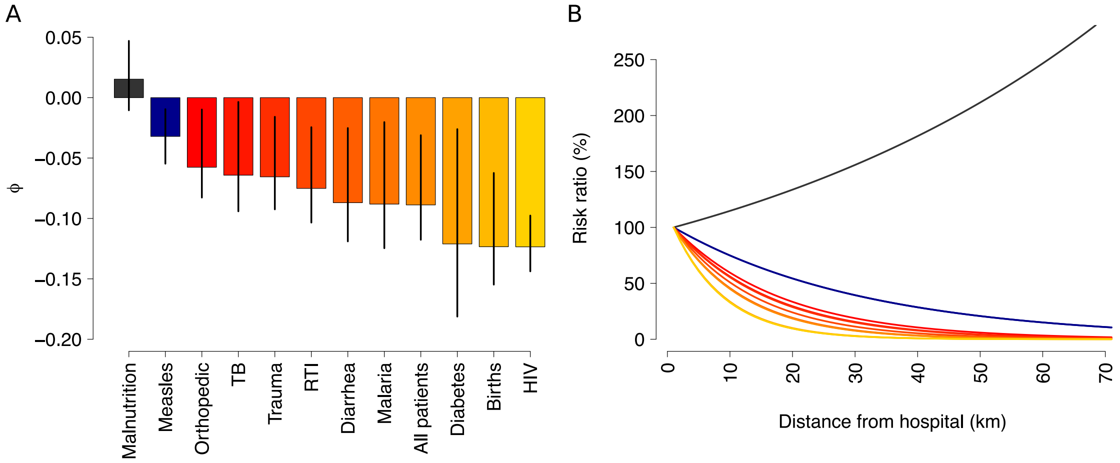

Figure S11. A) Estimated values of the decay/increase rate (i.e. ϕ) with distance for the logarithm of the RR of being hospitalized for different illness conditions and B) the resulting estimated RR as a function of distance (i.e. $\mathrm{RR(d)} = e^{\phi d}$).

8. References

[1] Population Division of the Department of Economic and Social Affairs of the United Nations Secretariat. Revision of World Population Prospects 2015; https://esa.un.org, accessed Jan 2017.

[2] Mitiku K, Bedada T, Masresha BG, Kegne W, Nafo-Traoré F, Tesfaye N, Yigzaw A. Progress in measles mortality reduction in Ethiopia, 2002–2009. *J Infect Dis* 2011; *204*(suppl 1): S232-S238.

[3] Prem K, Cook AR, Jit M, Projecting social contact matrices in 152 countries using contact surveys

and demographic data. PLOS Comput Biol 2017; 13(9): e1005697.
